# Whole genome-wide transcript profiling to identify differentially expressed genes associated with seed field emergence in two soybean low phytate mutants

**DOI:** 10.1186/s12870-016-0953-7

**Published:** 2017-01-18

**Authors:** Fengjie Yuan, Xiaomin Yu, Dekun Dong, Qinghua Yang, Xujun Fu, Shenlong Zhu, Danhua Zhu

**Affiliations:** Institute of Crop Science and Nuclear Technology Utilization, Zhejiang Academy of Agricultural Sciences, Hangzhou, 310021 China

**Keywords:** Low phytate seed, Soybean, Germination, Field emergence, Transcript

## Abstract

**Background:**

Seed germination is important to soybean (*Glycine max*) growth and development, ultimately affecting soybean yield. A lower seed field emergence has been the main hindrance for breeding soybeans low in phytate. Although this reduction could be overcome by additional breeding and selection, the mechanisms of seed germination in different low phytate mutants remain unknown. In this study, we performed a comparative transcript analysis of two low phytate soybean mutants (TW-1 and TW-1-M), which have the same mutation, a 2 bp deletion in *GmMIPS1*, but show a significant difference in seed field emergence, TW-1-M was higher than that of TW-1 .

**Results:**

Numerous genes analyzed by RNA-Seq showed markedly different expression levels between TW-1-M and TW-1 mutants. Approximately 30,000–35,000 read-mapped genes and ~21000–25000 expressed genes were identified for each library. There were ~3900–9200 differentially expressed genes (DEGs) in each contrast library, the number of up-regulated genes was similar with down-regulated genes in the mutant TW-1and TW-1-M. Gene ontology functional categories of DEGs indicated that the ethylene-mediated signaling pathway, the abscisic acid-mediated signaling pathway, response to hormone, ethylene biosynthetic process, ethylene metabolic process, regulation of hormone levels, and oxidation-reduction process, regulation of flavonoid biosynthetic process and regulation of abscisic acid-activated signaling pathway had high correlations with seed germination. In total, 2457 DEGs involved in the above functional categories were identified. Twenty-two genes with 20 biological functions were the most highly up/down- regulated (absolute value Log2FC >5) in the high field emergence mutant TW-1-M and were related to metabolic or signaling pathways. Fifty-seven genes with 36 biological functions had the greatest expression abundance (FRPM >100) in germination-related pathways.

**Conclusions:**

Seed germination in the soybean low phytate mutants is a very complex process, which involves a series of physiological, morphological and transcriptional changes. Compared with TW-1, TW-1-M had a very different gene expression profile, which included genes related to plant hormones, antioxidation, anti-stress and energy metabolism processes. Our research provides a molecular basis for understanding germination mechanisms, and is also an important resource for the genetic analysis of germination in low phytate crops. Plant hormone- and antioxidation-related genes might strongly contribute to the high germination rate in the TW-1-M mutant.

**Electronic supplementary material:**

The online version of this article (doi:10.1186/s12870-016-0953-7) contains supplementary material, which is available to authorized users.

## Background

Seeds are important for the survival and evolutionary success of plants and development of human cultures. Their germination traits are traditional agronomic traits and important for crop evolution and development [1, 2]. For soybean breeding and production, low seed germination percentage would decrease the density of soybean seedlings and ultimately affect the yield. Thus, the seed germination percentage and speed should not be negatively affected when developing any ecological, agronomic or nutritional traits.

Lowering the phytate content in crop seeds will be beneficial to improve seed nutritional traits and decrease water phosphorus level [3–5]. Therefore, there is a considerable interest in generating crops in which phytate synthesis is disrupted during seed development [6]. Seed phytate content can be eliminated by mutation or insertion of transgenes. Many low phytic acid (LPA) mutants have been created in different crops such as rice, maize, soybean and wheat [7–11]. However, some unwanted traits appeared in these mutants, which hindered the utilization of LPA mutants in crop breeding. Most primary LPA mutants often feature inferior grain yields, reduced seed viability or lower field emergence compared with their respective wild-type parents. Therefore, further improvement is needed before new LPA crops can be put into practical use [12–16]. For example, both laboratory and field observations demonstrated that the primary LPA rice mutant lines had lower grain yields and reduced seed viability compared with their respective parental lines [17, 18]. The extensive efforts are still needed in breeding LPA rice cultivars with competitive yields. Some undesirable agronomic and quality traits were also reported in several LPA soybean lines, particularly a lower rate of field emergence [8, 13, 14]. The breeding of high yield and LPA soybean varieties has been hindered by the inherent defects in the LPA mutations. Improving the seed germination trait is an important goal in breeding LPA soybean varieties. However, grain yield and field emergence can be improved through breeding and selection in soybean [15], and indeed, one LPA barley cultivar, CDC Lophy-1 (http://www.inspection.gc.ca/english/plaveg/pbrpov/cropreport/bar/app00006337e.shtml), has already been released for commercial production [16].

We previously developed two LPA mutants in soybean, which involved two non-allelic genes. The LPA traits of the mutant *Gm-lpa*-TW-1 were due to a 2-bp deletion in the *MIPS1* gene; inositol phosphate kinase (*GmIPK1*) was the mutation’s candidate gene which was related to the low phytate trait in *Gm*-*lpa*-ZC-2 mutant. Unlike other LPA mutants, *Gm-lpa*-ZC-2 appeared to have excellent seed viability (both germination and field emergence) [8]. However, the mutant *Gm-lpa*-TW-1 revealed a very low field emergence rate, especially in the spring season in Hangzhou, China [8]. Additionally, the seed germination speed decreased quickly during seed storage (unpublished data). Fortunately, an individual plant, which harbored a natural variation and had a significantly higher rate of field emergence, was found among the *Gm-lpa*-TW-1 lines. According to the results of the *MIPS1* gene sequence analysis, the individual has the same mutation site (2 bp deletion in *MIPS1*) as the *Gm-lpa*-TW-1 mutant (unpublished data), and it was named *Gm-lpa*-TW-1-M.

Seed germination is a complex process that includes imbibition, stirring and germination stages, which involve a series of physiological, morphological and transcriptional changes [19]. Several large-scale –omics methods, including transcriptomic, proteomic, and metabolomic methods, have been recently established to investigate the mechanisms of seed germination [20]. The great achievements in soybean genomics have led to application of large-scale gene expression analysis at both mRNA and protein levels to uncover the features of soybean traits. For instance, 69,338 distinct transcripts from 32,885 annotated genes were expressed in soybean seeds which from nine lines varying in oil composition and total oil content [21]. Until now, little is known about the mechanisms responsible for the low seed germination rates in soybean LPA mutants. Although we discovered a soybean LPA mutant with a higher rate of field emergence, seed germination trait is a comprehensive characteristic affected by many factors, including intrinsic and environment cues, during seed developmental and storage stages [22], which makes the genetic analysis of seed germination very difficult. Due to the development of high-throughput deep sequencing approaches, a new method regarding the relationships between gene expression profiles and gene functions has emerged. These technologies are useful for estimating overall gene expression profiles at different developmental stages and/or in different tissues. Although the biochemical pathways that affect seed germination are well characterized, there is still no integrated model describing the differentially expressed genes (DEGs) involved in soybean seed germination, in particular those used in soybean LPA mutant seed germination. The target of this research was to evaluate a large amount of cDNA sequence data, study seed germination trait in detail, and identify candidate genes that could be responsible for LPA soybean germination.

In this study, we used Illumina sequencing to investigate gene expression in soybean LPA mutant seeds at different germination stages and compared transcript reads with the most recent release of the *G. max* genome sequence (assembly Glyma 1.01).

## Methods

### Plant material and seed production

Two LPA soybean mutant lines, *Gm-lpa*-TW-1 (TW-1), *Gm-lpa*-TW-1-M (TW-1-M) and their wild-type parent Taiwan 75 were used in this experiment to evaluate the seed germination trait. Taiwan 75 is a vegetable soybean variety widely grown in Zhejiang Province. TW-1 was developed using gamma irradiation of wild type Taiwan 75, and TW-1-M was a natural mutant of the TW-1 line. Both TW-1 and TW-1-M had the same phytate content level and mutation site (2-bp deletion in *GmMIPS1*, unpublished data). Seed samples used for the germination evaluation were harvested from plants grown in neighboring plots in the same field. The seeds were produced in the 2012 spring season in Hangzhou, Zhejiang in the fields of the Experimental Farm of the Zhejiang Academy of Agricultural Sciences.

For differential gene expression detection, we used the two LPA mutants TW-1 and TW-1-M. To better understand and compare the expression differences of mutants TW-1 and TW-1-M during germination, three different germination stages were used in the analysis. These three stages included: the first one is imbibed seeds stage (about 24 h after seeds soaked, named TW-1-1 and TW-1-M-1), second stage is metabolism reactivation phase (about 30 h after seeds soaked), between seed imbibition and radicle emergernce (named TW-1-2 and TW-1-M-2) and the last stage is emergence of primary root which reached 1 mm in length (about 36 h after seeds soaked, named TW-1-3 and TW-1-M-3), three replicates were performed to construct eighteen DGE libraries, they were TW-1-1-1, TW-1-1-2, TW-1-1-3, TW-1-2-1, TW-1-2-2, TW-1-2-3, TW-1-3-1, TW-1-3-2, TW-1-3-3, TW-1-M-1-1, TW-1-M-1-2, TW-1-M-1-3, TW-1-M-2-1, TW-1-M-2-2, TW-1-M-2-3, TW-1-M-3-1, TW-1-M-3-2 and TW-1-M-3-3 . These three stages based on the three phases of germination process (fast water uptake, metabolism reactivation and radicle emergence) were chosen for study according to our germination experiments (as shown below) and some reports [19, 23].

### Germination experiments

Two LPA soybean mutant lines and their wild-type parents were used for germination experiments that included two treatments, warm germination and accelerated aging tests. The method was from Meis et al. with a slight modification [13]. For the warm germination, 100 seeds of each line were planted in a Petri dish containing B5 agar gel (50 seeds per 15-mm Petri dish) and were placed in a 25 °C germination chamber in the dark for 4 d. The lines used to evaluate the effectiveness of accelerated aging tests for predicting field emergence after long time storage included the two LPA mutants (TW-1 and TW-1-M) and their wild-type variety Taiwan 75. In total, 200 seeds from each line were placed over 400 ml of distilled water in an acrylic box and covered. The boxes were placed in a chamber at 40 °C for 96 h. The samples were removed from the chamber and planted in the same manner as those from the warm germination. Seed germination in the Petri dishes was defined as the point at which the radical pierced the seed coat. In total, 100 seeds were used per line, per treatment, and three replicates were performed. The experiments were organized in a randomized complete-block design, and the data for each germination test were analyzed by the linear model procedure of SAS statistical software (release 8.02).

### Total RNA isolation

Samples from three germination stages were used in this research. Total RNA was isolated using an E.Z.N.A. plant RNA kit (Omega Bio-tek, Inc., USA) according to the manufacturer’s protocol. Genomic DNA contamination was eliminated by RQ1 RNase-Free DNase (Promega, USA).

### cDNA library construction and sequencing

The quality of total RNA (OD260/280 = 1.8 ~ 2.2, 28 s/18 s >1.8, and RIN >8) was assessed by using a 2100 Bioanalyzer (Agilent, Santa Clara, CA, USA) and checked using agarose gel electrophoresis. rRNAs were then removed from the total mRNAs in accordance with the instructions included with the Ribo-Zero™ rRNA Removal Kit (Plant Seed/Root) (Epicentre, Madison, WI, USA), final concentration of all RNA samples was adjusted to 500 ng/μl after quantification. cDNA libraries were prepared with the SMART™ cDNA Library Construction Kit, Takara Biomedicak Technology (Beijing) Co.,Ltd and 140–220 bp paired-end reads were generated on the Illumina HiSeq 2000 platform. (Illumina, USA). RNA sequencing was performed by staff at Zhejiang Tiank (Hangzhou, China).

### Differential expressed gene detection

Sequencing-received raw image data is transformed by base calling into sequence raw data, and is stored in FASTQ format. All the raw data described in the research from eighteen libraries were published in SRA database. The raw data were filtered by Trimmomatic software to remove adaptor reads, low quality reads (reads containing unknown nucleotides “Ns”), reads of copy number = 1 and reads of lengths less than 20 bp, yielding a dataset consisting of clean reads. For the annotation of reads, clean reads were mapped to the soybean database using software TopHat [24]. Mismatches of no more than two bases were allowed in the alignment. The number of clean reads for each gene was calculated and normalized using a variation of the fragment/Kb/million (FPKM) method. The FPKM method corrects for biases in total gene exon size and normalizes for the total fragment sequences obtained in each tissue library with bioconductor software:cuffquant and cuffnorm. In this experiment, we removed low expression genes which value of FPKM <1 in any library as threshold to count the expressed genes. Principal component analysis between different libraries about their gene expression datasets was carried out by R language software package http://factominer.free.fr/. This method used variance-stabilized data to obtain sample-to-sample distance.

For the tissue-specific analyses, in order to identify differentially expressed genes, cuffdiff software in software packages cufflinks were used to perform pairwise comparisions of stages, and for packages with a corrected *P*-value of 0.05 and a Log2-fold change [24]. Genes with a *P*-value < 0.05 and estimated absolute Log2-fold change >1 in sequence counts across libraries were considered to be significantly differentially expressed.

GO enrichment analysis was performed using the SmartGo tool (a software package developed by Tianke company China), we using a hypergeometric (Fisher’s exact test) test to map all DGEs to terms in the Go database (http://www.geneontology.org) to look for significantly enriched Go terms in DGEs comparing to the genome background. The *P*-value is corrected by Bonferroni, we chose a *p* value <0.05 as the threshold value. The GO term (*P* < 0.05) is defined as significantly differentially expressed genes enriched GO term. Pathway enrichment analysis method is the same as that used in the GO analysis. Fisher test was used to check up enrichment gene KEGG pathway with corrected *P*-value of 0.05.

### Quantitative real-time PCR (qRT-PCR)

To validate the data obtained by Illumina RNA-seq, qRT-PCR was performed on 10 genes with log2FC ratios ranging from 2 to 11. At first, we selected *ACT11、TUA* and *CYP2* three housekeeping genes to analyze the stability of their expression using geNorm software (v3.50). The relatively most stable housekeeping gene *ACT11* was used to normalize expression levels of selected genes. The RNA samples used for the qRT-PCR assay were the same as those used for the DEG experiments. SYBR Green Real time PCR Master Mix (TOYOBO Biotech Co., Ltd) was used on a Roche (LightCycler®^480^, USA) instrument according to the manufacturer’s instructions. Each 20-μl reaction comprised a 2-μl template, 10-μl SYBR Green Realtime PCR Master Mix-Plus, 1.2 μl (10 μM) of each primer, 2-μl Plus Solution and 3.6-μl ddH2O. The quantification of gene expression levels was performed in triplicate using the corrected relative –2^ΔΔCT^ method by comparing the data with the internal control gene *Act11* [25]. qRT-PCR efficiency was determined by five serial five-fold dilutions of cDNA, and the standard curve confirmed them at high efficiency rates. All primers used for qRT-PCR amplification were designed by Primer Premier 5 and according to the gene mRNA sequence from http://www.ncbi.nlm.nih.gov/genbank/.Primers were synthesized in Shanghai Sangon Biological Engineer Technology and Services Co., Ltd. (Shanghai, China) and are given in Additional file 1.

## Results

### Seed germination of different soybean mutants

To explore seed germination trait between the mutants and their wild-type parents, we evaluated the germination percentage and speed of soybean lines under both warm germination and accelerated aging test conditions. The goal of the accelerated aging test was to identify the rate at which percent germination declines of soybean lines. The lines that performed well in the accelerated aging test would be expected to maintain viability under prolonged storage.

**Fig. 1:**
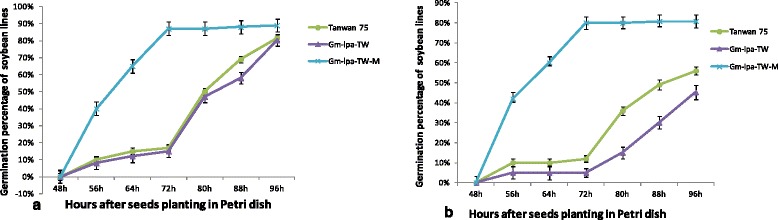
Changes in germination percentages and germination speed of LPA mutants and their wild-type parents. **a** germination percentages during warm germination test; **b** germination percentages during accelerated aging test. In both the warm germination test and accelerating aged test, the mutant TW-1-M performed well, with a high germination percentage (more than 80%) and speed, compared with the TW and wild-type Taiwan 75

There were statistically significant differences in the germination speed between TW-1-M and TW-1 in both warm germination and accelerated aging tests (Fig. 1). TW-1-M cost about 72 h to reach the highest germination percentage, whereas TM-1 needed more than 96 h to reach its highest germination percentage point. The germination speeds of TW-1 and Taiwan 75 were the same in both accelerated aging and warm germination tests.

In the warm germination test, the germination percentage in TW-1 was very similar to that of its wild-type parent Taiwan 75. The final germination percentage was about 80% according to the germination curve. The germination percentage of TW-1-M was slightly higher than those of the other two lines. The final germination percentage was about 85% (Fig. 1). There was no statistically significant difference between TW-1-M and TW-1. In the accelerated aging test, TW-1-M showed a very good performance in the germination percentage (about 80%). The TW-1 line performed a very low germination percentage (about 45%) and the germination percentage of Taiwan 75 was about 50% (Fig. 1).

The seed germination trait of TW-1-M performed better than those of TW-1 and Taiwan 75. This result indicated that the mutant TW-1-M, with the same mutation site as TW-1, will have significantly better germination trait and storage stability. This result was confirmed by the seed field emergence rate of three lines. The seed field emergence rate of TW-1-M (about 50%) was significant higher than that of TW-1 and Taiwan 75 (less than 10%) after 2-year storage at room temperature.

### Digital gene expression (DGE) library sequencing

To characterize gene expression profiles during soybean germination, the high-through put read sequencing analysis of soybean seedling libraries were performed using the Illumina RNA-sequencing analyzer platform. The differences in the gene regulatory pathways between the two LPA mutants were analyzed at the three seed germination stages. The 18 DGE libraries were sequenced and generated approximately 802 million raw reads. All raw data had been published in GEO with accession number GSM2195640 (TW-1-1-1), GSM2195641 (TW-1-1-2), GSM2195642 (TW-1-1-3), GSM2195643 (TW-1-2-1), GSM2195644 (TW-1-2-2), GSM2195645 (TW-1-2-3), GSM2195646 (TW-1-3-1), GSM2195647 (TW-1-3-2), GSM2195648 (TW-1-3-3), GSM2195649 (TW-1-M-1-1), GSM2195650 (TW-1-M-1-2), GSM2195651 (TW-1-M-1-3), GSM2195652 (TW-1-M-2-1), GSM2195653 (TW-1-M-2-2), GSM2195654 (TW-1-M-2-3), GSM2195655 (TW-1-M-3-1), GSM2195656 (TW-1-M-3-2) and GSM2195657 (TW-1-M-3-3). After filtering the low quality reads, the total number of clean reads in each library ranged from 37.7 to 62.1million (Table 1). The percentage of clean reads among the raw reads in most libraries were more than 95%.

**Table 1:** Library read analyses statistics

	Total raw reads	Total clean reads	Valid ratio	Gene mapped reads	Gene mapped ratio	Gene unique mapped reads	Gene unique mapped ratio	Non-splice reads ratio	Splice reads ratio	Total reads mapped expressed genes
TW-1-1-1	54846496	53989364	98.44%	47980066	88.87%	40357180	84.11%	55.68%	28.44%	31987
TW-1-1-2	61454226	60684460	98.75%	54536489	89.87%	45869439	84.11%	55.98%	28.13%	32935
TW-1-1-3	46813834	45625642	97.46%	40571957	88.92%	32364788	79.77%	53.88%	25.89%	32613
TW-1-2-1	42866726	41833914	97.59%	36435236	87.09%	29690167	81.48%	54.55%	26.93%	32799
TW-1-2-2	40017620	39514560	98.74%	35947369	90.97%	29879201	83.12%	53.91%	29.21%	33015
TW-1-2-3	44069752	43420982	98.53%	39540531	91.06%	31212328	78.94%	51.35%	27.59%	33479
TW-1-3-1	43704478	42954548	98.28%	38687168	90.07%	31702965	81.95%	53.53%	28.42%	30656
TW-1-3-2	49604414	48907380	98.59%	44561575	91.11%	36044941	80.89%	49.61%	31.28%	33848
TW-1-3-3	44165126	41611906	94.22%	35653176	85.68%	28482253	79.89%	49.44%	30.45%	34820
TW-1-M-1-1	54461020	53698832	98.60%	49119558	91.47%	40110386	81.66%	55.04%	26.61%	32330
TW-1-M-1-2	46244798	45634652	98.68%	41698366	91.37%	35054413	84.07%	55.21%	28.85%	31706
TW-1-M-1-3	53416206	52732078	98.72%	48443543	91.87%	39005105	80.52%	51.91%	28.61%	32999
TW-1-M-2-1	51359166	50426196	98.18%	46437117	92.09%	38158539	82.17%	51.46%	30.71%	33151
TW-1-M-2-2	62294034	61477542	98.69%	56456407	91.83%	46353592	82.11%	50.80%	31.31%	33871
TW-1-M-2-3	43755042	42995004	98.26%	39690338	92.31%	32335903	81.47%	50.00%	31.47%	32406
TW-1-M-3-1	62931516	62062754	98.62%	56549018	91.12%	45018182	79.61%	50.79%	28.82%	34135
TW-1-M-3-2	39450214	37671510	95.49%	32733417	86.89%	25369978	77.50%	49.32%	28.18%	34345
TW-1-M-3-3	46654868	45873652	98.33%	42141833	91.87%	35304742	83.78%	52.61%	31.16%	33738

### Mapping reads to the reference transcriptome

Among the clean reads, 88.9–92.3% of transcripts from the TW-1 and TW-1-M mutants were perfectly mapped to the soybean reference genome. The number of unique reads mapped to genes was from 25.4 to 46.4 million. The percentage of these unique reads was about 80%. The number of read-mapped genes ranged from 30,656 to 34,820 (Table 1). The TW-1-M-3 library contained the highest number of read-mapped genes, whereas the TW-1-M-1 library contained the lowest number of read-mapped genes. These data suggested that more genes were expressed in the TW-1-M-3 library compared with the other five libraries.

### Variation in gene expression levels quantified by DGE profiles in the LPA mutants

**Fig. 2:**
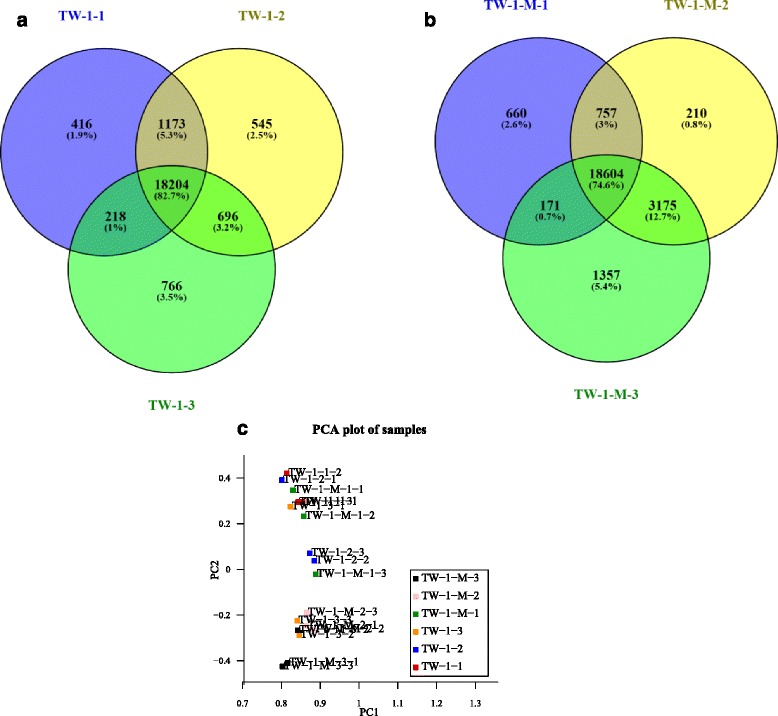
Venn diagrams showing the overlapping of expressed genes in LPA mutants. **a** Venn diagram showing the overlaps in expressed genes among seed germination stages of the TW-1 mutant. **b** Venn diagram showing the overlaps in expressed genes among seed germination stages of the TW-1-M mutant. **c** PCA analysis plots for TW-1-M and TW-1 samples. Principal component analysis for the TW-1-M suggests that gene expression in TW-1-3 and TW-1-M-2 is overall similar to each other, TW-1-1 and TW-1-M-1 also have same gene expression pattern. TW-1-2 and TW-1-M-3 perform special gene expression comparing with other four simples, and they also have different gene expression pattern with each other

Based on the deep sequencing of the 18 DGE libraries, the number of clean reads in each library was normalized to the FPKM to obtain the normalized gene expression level. Each mapped soybean gene with FPKM value in each of the 18 libraries was listed in the Additional file 2. Twenty thousand eleven (42.7% of reference genes in soybean) and 20,192 (43.1%) expressed genes were detected in the LPA mutants TW-1-1 and TW-1-M-1, respectively. In total, 20,618 (44.0% of reference genes in soybean) and 22,746 (48.5%) expressed genes were detected in the mutants TW-1-2 and TW-1-M-2. Additionally, 19,884 (42.4% of reference genes in soybean) and 23,307 (50.0%) expressed genes were detected in the TW-1-3 and TW-1-M-3. A total of 22,018 genes were expressed in the LPA TW-1 mutants during the whole germination process. Eighteen thousand two hundred four were constitutively expressed, 1727 were stage-specific and 2087 were expressed at the two stages (Fig. 2a). During the seed germination process of TW-1-M, 24,934 genes were expressed. Eighteen thousand six hundred four were constitutively expressed, 2227 were stage-specific and 4103 were expressed at the two stages (Fig. 2b). The number of stage-specific expressed genes in mutant TW-1- 3 was significantly more than in other TW-1 mutants, indicating that TW-1- 3 expressed more specific genes that were related to seed germination. TW-1-M-3 has the most specific and total number of genes expressed compared with the other TW-1-M and TW-1 mutants, and this increased transcript diversity in TW-1-M-3 indicates that it might express a distinctive suite of genes for cellular functions, which may be vital for seed germination.

Furthermore, we analyzed the relationship of different samples with principal component analysis between experiments about their gene expression datasets. Principal component analysis suggested that the gene expression in TW-1-3 and TW-1-M-2 is overall similar to each other. TW-1-1 and TW-1-M-1 also had the same gene expression pattern. TW-1-2 and TW-1-M-3 performed special gene expression compared with other four simples and had different gene expression with each other (Fig. 2c).

### Screening of DEGs from massive datasets

To identify and compare the DEGs in LPA mutants during the different germination stages, we used cufflinks software packages to perform pairwise comparisons of DGE libraries between two LPA mutants (TW-1-1 vs.TW-1-M-1, TW-1-2 vs. TW-1-M-2 and TW-1-3 vs.TW-1-M-3). To judge the significance of differences in expressed genes, we used two criteria: *P*-value < 0.05 and absolute Log2-fold change >1.

In total, there were 3677 DEGs at each germination stage in the LPA mutants (TW-1 and TW-1-M). Among these genes, 3099 (84%) were up-regulated and 548 (16%) were down-regulated in TW-1-M compared with TW-1 (Additional file 3). We found that 1354 genes were down-regulated and 2596 genes were up-regulated in TW-1-M -1compared with TW-1-1. In total, 5060 genes were up-regulated and 4185 genes were down-regulated in TW-1-M-2 compared with TW-1-2. Additionally, 3395 genes were down-regulated and 1983 genes were up-regulated in TW-1-M-3 compared with TW-1-3 (Additional file 3, Fig. 3).

**Fig. 3:**
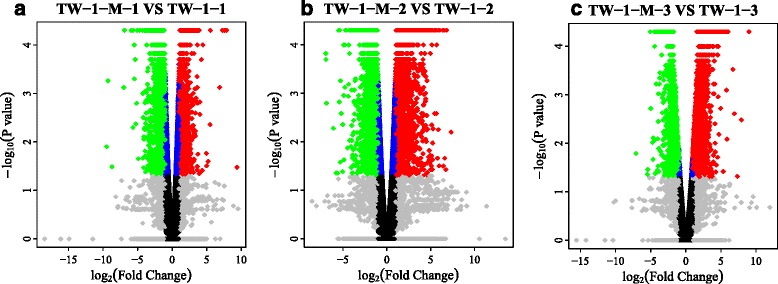
Cufflinks volcano plots of differentially express gene. **a**-**c** Cufflinks valcano plots for each contrast library showing variances in gene expression with respect to fold-change and significance. Each *dot* represents an individual gene. *Black dots* represent genes with no significantly differentially expressed, *green dots* represent significant down-regulated DEGs and *red dots* represents significant up-regulated DEGs

**Fig. 4:**
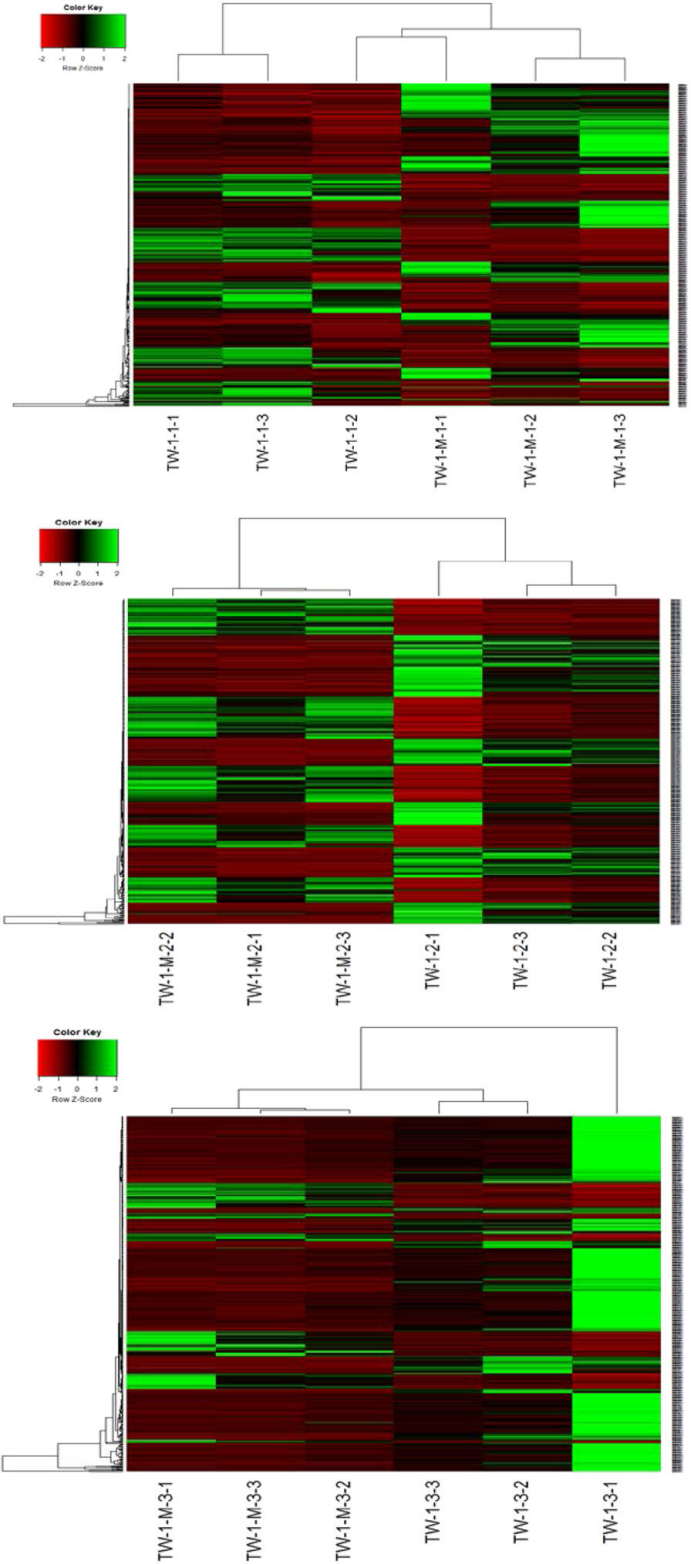
Heat map generated from the top 500 DEGs as reported by heatmap.2 in R language. The *red* color indicated higher levels of gene expression while *green* indicated lower expression by log_10_ (FPKM). The results showed that expression level of 500 DEGs inTW-1-M-2 were very different from TW-1-2, on the contrary, many top DEGs’ expression level were similar with each other between TW-1-M-3 and TW-1-3 libraries

To illustrate differences between different libraries, heat maps were constructed using heatmap.2 software showing log_10_ (FPKM) expression values for top 500 of the most differentially expressed genes in six contrast libraries. The results showed that expression level of the top DEGs in TW-1-M-1 was different from TW-1-1 (Fig. 4a). Furthermore, expression level of 500 DEGs in TW-1-M-2 was different from TW-1-2. Most of genes showed completely contrary expression level in these two contrast libraries (Fig. 4b). We concluded that TW-1-M-2 was different from TW-1-2 during seed germination. On the contrary, the expression levels of many top DEGs were similar with each other between TW-1-M-3 and TW-1-3 libraries (Fig. 4c). It should be noted that the expression level of DEGs which related with germination in these two mutants becomes similar in this germination stage.

### Further analysis of DEGs between the two genotypes

Based on the results of the two mutants, DEGs were further compared between TW-1 and TW-1-M. In total, 190 genes were expressed only in the TW-1-1 vs TW-1-M-1 library and most of them were up-regulated in TW-1-1; 616 genes were found in the TW-1-2 vs TW-1-M -2 library and 65.6% of them were up-regulated in TW-1-M-2; 169 genes were specifically expressed in the TW-1-3 vs TW-1-M-3 library and 67.5% of them were up-regulated in TW-1-M-3 (Additional file 4). These genes might have special functions leading to different seed germination traits.

### GO functional enrichment analysis of DEGs in the different libraries from LPA mutant genotype

GO encompasses three domains: cellular component, biological process and molecular function. The basic GO unit is the GO term. Every GO term belongs to a particular category. GO terms with Bonferroni-corrected *P*-values < 0.05 were defined as being significantly enriched in DEGs.

**Fig. 5:**
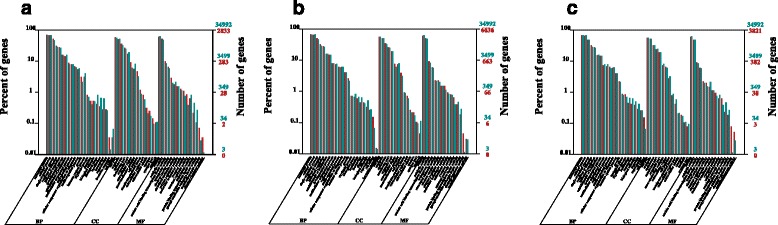
Functional categorization of significantly DEGs during the seed germination stage. **a** Functional categorization of significantly DEGs between TW-1-M-1 and TW-1-1. **b** Functional categorization of significantly DEGs between TW-1-M-2 and TW-1-2. **c** Functional categorization of significantly DEGs between TW-1-M-3 and TW-1-3

In our study, most DEGs regardless of regulation direction from different libraries were involved in the categories of nucleus (GO:0005634), cell part (GO: 0044464), plastid (GO:0009536), membrane (GO:0016020) and intergral component of membrane (GO:0016021) with respect to cellular components. Under the biological process, most of the DEGs could be divided into five categories, metabolic process (GO:0044260, GO:0044710 and GO:0019538), oxidation-reduction process (GO:0055114), response to environmental stimulus, plant hormone signaling pathway. With regard to molecular function, DNA, RNA and protein binding are the largest DEG categories, and oxidoreductase activity is also a very important functional group (Fig. 5, Additional file 5).

Seed germination is a complex process which involved in many activities of some key enzymes in glycolysis, pentose phosphate pathway, the tricarboxylicacid cycle, protein and lipid metabolism [19]. Furthermore, reactive oxygen species production including the superoxide anion radical, hydrogen peroxide and the hydroxyl radical can cause oxidative damage to cellular components and reduce seeds ability to germination [26, 27]. Finally, plant hormones such as abscisic acid, gibberellins and ethylene play a very important role in seeds germination [28]. According to above researches and the DGEs numbers in functional categories, in this research, we were concerned with the functional categories of DEGs (regardless of regulation direction) related to seed germination process. In the contrasting groups TW-1-1 and TW-1-M-1, the DEGs most possibly related to seed germination were from the biological process category: oxidation-reduction process (GO:0055114), protein metabolic process (GO:0019538), carbohydrate metabolic process (GO:0005975), lipid metabolic process (GO:0006629) and hormone transport (GO:0009914). Biological processes, which would be responsible for seed germination in contrasting groups TW-1-2 and TW-1-M-2 were classified in oxidation-reduction (GO:0055114), protein metabolic process (GO:0019538), lipid metabolic process (GO:0006629), response to hormone (GO:0009725), regulation of hormone levels (GO:0010817) and carbohydrate metabolic process (GO:0005975). We also found some biological processes involved with seed germination in the TW-1-3 and TW-1-M-3 groups, including the oxidation-reduction process (GO:0055114) and seed germination (GO:0009845). These biological processes might be highly related to seed germination traits (Fig. 5, Additional file 5).

### Pathway enrichment analysis of DEGS

A pathway enrichment analysis is an effective method to elucidate DGE biological functions. A pathway-based analysis can identify significantly enriched metabolic and signal transduction pathways in DEGs by comparing their whole-genome backgrounds [29]. The formula used for this calculation was essentially identical to that used in the GO analysis, with pathways having *P*-values < 0.05 being defined as significant DEGs.

**Fig. 6:**
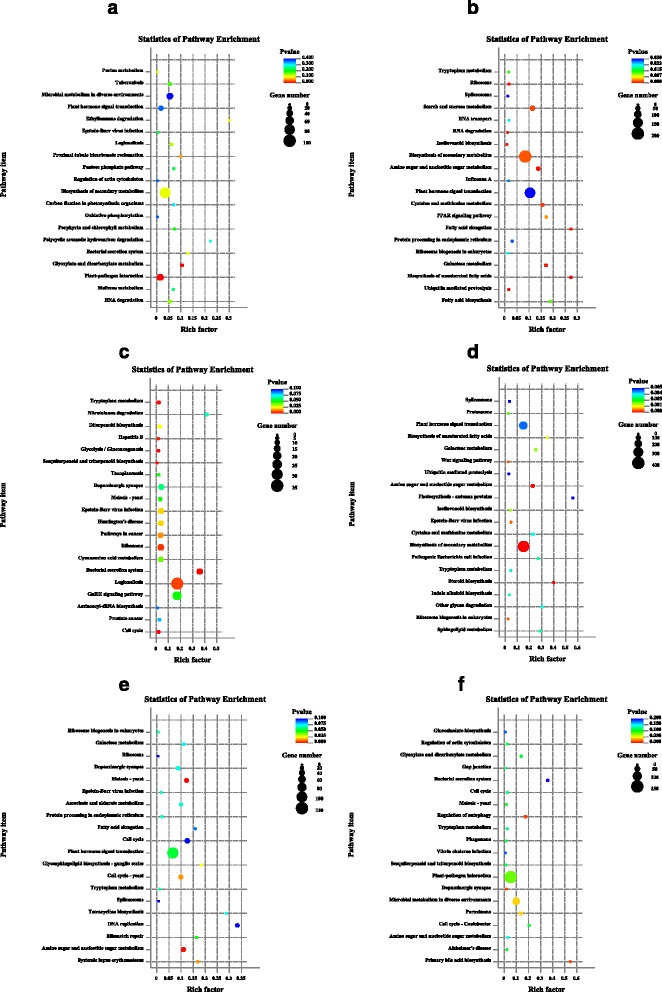
Scatter diograms illustrating the pathway enrichment analysis. **a** down-regulated enrichment pathway items in TW-1-M-1 relative to TW-1-1. **b** up-regulated enrichment pathway items in TW-1-M-1 relative to TW-1-1. **c** down-regulated enrichment pathway items in TW-1-M-2 relative to TW-1-2. **d** up-regulated enrichment pathway items in TW-1-M-2 relative to TW-1-2. **e** down-regulated enrichment pathway items in TW-1-M-3 relative to TW-1-3. **f** up-regulated enrichment pathway items in TW-1-M-3 relative to TW-1-3

DEGs in 80 metabolic and signal transduction pathways were found between TW-1-1 and TW-1-M-1 contrast libraries. The mainly regulated pathways with the most up-regulated gene numbers in TW-1-M-1 were ‘biosynthesis of secondary metabolites’, ‘plant hormone signal transduction’, ‘Ascorbate and aldarate metabolism’ and ‘starch and sucrose metabolism’. There were 113 enrichment pathways involved in the TW-1-2 and TW-1-M-2 contrast libraries. Among these pathways, six pathways might be related to seed germination, ‘biosynthesis of secondary metabolites’, ‘starch and sucrose metabolism’, ‘flavone and flavonol biosynthesis’, ‘isoflavonoid biosynthesis’ and ‘plant hormone signal transduction’ and ‘gulutathione metabolism’. These pathways were up-regulated in TW-1-M-2. The most enriched pathways responsible for seed germination in the TW-1-M-3 vs TW-1-3 contrast libraries were ‘plant hormone signal transduction’ and ‘starch and sucrose metabolism’. These two pathways performed two contrast regulation directions, some genes were up-regulated and the others were down-regulated (Fig. 6, Additional file 6).

### DEGs analysis in each category regarding seed germination-related biological processes in the LPA mutant TW-M

GO functional annotations and a pathway enrichment analysis of DEGs (regardless of directions) in the high germination mutant implied that the DEGs in the most highly enriched biological processes and pathways were most likely contributing to the good seed germination trait. Because we are interested in seed germination-related biological processes, we focused on the DEGs involved in pathways and functional categories related to seed germination biological processes. All of these DEGs are listed in Additional file 7.

In total, 527 DEGs in the TW-1-1 and TW-1-M-1 contrast libraries were related to seven different biological processes. Among these genes, 97 DEGs were down-regulated and 213 DEGs were up-regulated in oxidation-reduction process in mutant TW-1-M-1, other 384 DGEs were all up-regulated in TW-1-M-1 in hormone-mediated signaling pathway, auxin-activated signaling pathway, response to auxin, auxin transport, hormone transport, gibberellic acid mediated signaling pathway, gibberellin mediated signaling pathway and gibberellin biosynthetic process.

In total, 1240 DEGs between the TW-1-2 and TW-1-M-2 contrast libraries could be separated into five functional categories, including the response to hormone, ethylene biosynthetic process, ethylene metabolic process, regulation of hormone levels, and oxidation-reduction process. Of these, 54 genes were up-regulated in the hormone biosynthetic process of TW-1-M-2 and 69 genes related to hormone metabolic process were also up-regulated in TW-1-M-2. The most DEGs were found in the oxidation-reduction process, with 408 up-regulated genes in TW-1-M-2. In total, 880 down-regulated genes were found in TW-1-M-2 mutant in ten different functional categories, including hormone-mediated signaling pathway (225 DEGs), response to hormone (202 DEGs), response to abscisic acid (111 DEGs), ethylene-activated signaling pathway (83 DEGs), abscisic acid-activated signaling pathway (81 DEGs), response to ethylene (60 DEGs), ethylene biosynthetic process (36 DEGs), ethylene metabolic process (36DEGs), regulation of flavonoid biosynthetic process (27 DEGs) and regulation of abscisic acid-activated signaling pathway (19 DEGs).

The 690 DEGs in the TW-1-3 and TW-1-M-3 contrast libraries were divided into seven functional categories, including the hormone-mediated signaling pathway (178 DEGs), response to abscisic acid (78 DEGs), ethylene-mediated signaling pathway (65 DEGs), oxidation-reduction process (232 DEGs), abscisic acid-activated signaling pathway (59 DEGs), response to ethylene (47 DEGs) and seed germination (31 DEGs). Of these, all genes were down-regulated in the TW-1-M-3 mutant (Additional file 7).

### Possible DEGs for major roles in response to better seed germination trait

The 22 most DEGs (absolute value Log2FC >5) were identified by a DGE analysis of the TW-1-M (Additional file 8). Among them, 13 genes were up-regulated and nine genes were down-regulated. These included three transcription factors, one cytochrome gene, two auxin-induced protein genes, four oxidase genes, one isoflavone 7-0-methyltransferase gene, six genes related with carbohydrate metabolism, two genes which catalyzed the glutathione metabolism, one expansin gene and two other genes. The functional annotations of these genes are shown in Table 2. These genes might be the most important genes contributing to the high seed germination percentage and speed in TW-1-M.

**Table 2:** Most differentially expressed genes identified by DGEs analysis in TW-1-M relative to TW-1

Gene	Gene annotation	Regulation direction
gene21202	1-Cys peroxiredoxin	down
gene26324	auxin-induced protein 15A	up
gene30929	auxin-induced protein 15A-like	up
gene30772	cytochrome P450 CYP82D47-like	down
gene47663	expansin-A1-like	down
gene22557	F-box/kelch-repeat protein At3g23880-like	up
gene22518	GATA transcription factor 7-like	down
gene8527	glutathione synthetase, chloroplastic-like	down
gene23718	hydroquinone glucosyltransferase-like	up
gene52332	isoflavone 7-O-methyltransferase-like	up
gene35300	late embryogenesis abundant protein-like	down
gene30019	oxidoreductase	down
gene43660	polygalacturonase inhibitor-like	up
gene37153	polyphenol oxidase A1, chloroplastic-like	up
gene24050	probable F-box protein At4g22030	down
gene18907	probable glutathione S-transferase	up
gene36822	probable glycosyltransferase At5g03795	down
gene42818	reticuline oxidase-like protein	up
gene51756	soyasapogenol B glucuronide galactosyltransferase-like	up
gene53209	two-component response regulator ARR14-like	up
gene6319	UDP-glycosyltransferase 72D1-like	up
gene51015	UDP-glycosyltransferase 91A1-like	up

In this study, we also analyzed some DEGs with high FPKM values (FPKM value in library TW-1 or TW-1-M more than 100) (Additional file 9), these genes could be divided into eight groups. The first group contained 13 genes which mainly participated in carbohydrate metabolism (glycosyltransferase, glucanase, galactinol synthase). The second group was composed of 11 genes, they were one abscisic-acid-receptor, two auxin –regulated protein, six ethylene-responsive transcription factors, one gibberellin 2-beta-dioxygenase and one gibberellin-regulated protein. The third group contained 10 transcript factors. The fourth group were made up of nine oxidoreductases (three carboxylateoxidase, one acyl-CoA oxidase, one ascorbate peroxidase, one L—ascorbate oxidase, two peroxidase and one aldo-keto reductase). The fifth group constituted five glutathione S-transferases. The sixth group inculded four embryogenesis protein genes. The seventh group was made up three flavonol genes. The last group comprised two catalase genes (Table 3).

**Table 3:** DEGs with high FRPM values in TW-1 and TW-1-M

Gene	Gene_annotation	TW-1-M	TW-1	Regulation
gene13922	1-aminocyclopropane-1-carboxylate oxidase 1	1160.28	240.427	up
gene20644	1-aminocyclopropane-1-carboxylate oxidase 1-like	326.818	56.2496	up
gene20259	1-aminocyclopropane-1-carboxylate oxidase-like	446.817	204.574	up
gene2676	2-hydroxyisoflavanone dehydratase	148.017	308.306	down
gene34732	ABC transporter F family member 1, transcript variant X1	57.2258	240.966	down
gene37621	abscisic acid receptor PYL12-like	656.208	1350.98	down
gene12186	acyl-CoA oxidase	55.391	117.437	down
gene58187	ascorbate peroxidase 1, cytosolic	270.578	105.846	up
gene29377	auxin down-regulated protein	2132.82	759.573	up
gene37700	auxin-repressed 12.5 kDa protein-like, transcript variant X1	116.909	278.5	down
gene8950	catalase	62	192.975	down
gene14347	catalase	90.0193	255.074	down
gene7840	dolichyl-diphosphooligosaccharide--protein glycosyltransferase subunit 4A	236.779	110.339	up
gene54561	dolichyl-diphosphooligosaccharide--protein glycosyltransferase subunit 4A	223.879	104.715	up
gene27626	embryonic protein DC-8-like	44.7358	269.219	down
gene31117	endo-1,3-beta-glucanase	59.2309	540.571	down
gene37696	ethylene-responsive transcription factor 12	212.678	879.038	down
gene42281	ethylene-responsive transcription factor 12-like	374.843	794.408	down
gene15603	ethylene-responsive transcription factor ERF110-like	155.112	347.213	down
gene49187	ethylene-responsive transcription factor RAP2-1-like	56.2688	135.164	down
gene24409	ethylene-responsive transcription factor RAP2-3-like	668.14	1576.5	down
gene42969	ethylene-responsive transcription factor RAP2-3-like	1167.92	3510.15	down
gene35673	F-box protein SKP2B, transcript variant X1	77.6451	172.134	down
gene55554	flavonol synthase/flavanone 3-hydroxylase-like	84.0581	183.276	down
gene8335	galactinol synthase 1	500.381	1388.7	down
gene55115	galactinol synthase 2	23.0731	379.858	down
gene15912	galactinol--sucrose galactosyltransferase, transcript variant *X*2	142.3	396.706	down
gene11959	galactinol--sucrose galactosyltransferase-like	90.2918	344.135	down
gene42444	gibberellin 2-beta-dioxygenase-like	240.649	96.8673	up
gene52252	gibberellin-regulated protein 4-like	6582.18	2751.57	up
gene56620	glutathione S-transferase GST 18	74.9299	251.187	down
gene18908	glutathione S-transferase GST 7	223.983	96.0631	up
gene27455	glutathione S-transferase L3, transcript variant X1	158.692	512.444	down
gene44166	glutathione transferase	132.399	308.722	down
gene21642	homeodomain-leucine zipper protein 56, transcript variant X1	189.04	91.3283	up
gene46225	isoflavone 7-O-glucosyltransferase 1-like	30.5776	116.967	down
gene8972	L-ascorbate oxidase homolog	139.264	63.2909	up
gene27264	late embryogenesis abundant protein D-34-like	300.459	1242.39	down
gene35300	late embryogenesis abundant protein-like	328.87	2372.13	down
gene37327	late embryogenesis abundant protein-like	52.5198	587.359	down
gene30694	MYB transcription factor MYB50	142.722	41.363	up
gene9199	MYB transcription factor MYB68	52.8014	152.65	down
gene46117	peroxidase 12-like	192.985	80.4326	up
gene24224	peroxidase 15-like, transcript variant X1	179.556	36.2356	up
gene16824	probable aldo-keto reductase 2	42.1291	513.099	down
gene47762	probable galactinol--sucrose galactosyltransferase 6, transcript variant X1	161.157	60.6944	up
gene44169	probable glutathione S-transferase parC	86.7473	287.001	down
gene8658	putative F-box protein PP2-B12	77.8839	458.651	down
gene11905	RING-H2 finger protein ATL48	269.579	746.254	down
gene55008	stachyose synthase	57.1634	224.996	down
gene27675	transcription elongation factor 1 homolog	129.432	322.136	down
gene12634	transcription factor HEC1-like	80.3	168.632	down
gene30355	UDP-glucose 4-epimerase GEPI48-like, transcript variant X1	48.3533	148.505	down
gene52306	UDP-glucose 6-dehydrogenase 1	172.346	65.6367	up
gene22682	UDP-glucose dehydrogenase, transcript variant X1	181.645	84.1597	up
gene54704	UDP-glycosyltransferase 73C2-like	392.204	114.358	up
gene45	zinc finger CCCH domain-containing protein 20	97.306	265.596	down

### Confirmation of read-mapped genes by qRT-PCR

**Fig. 7:**
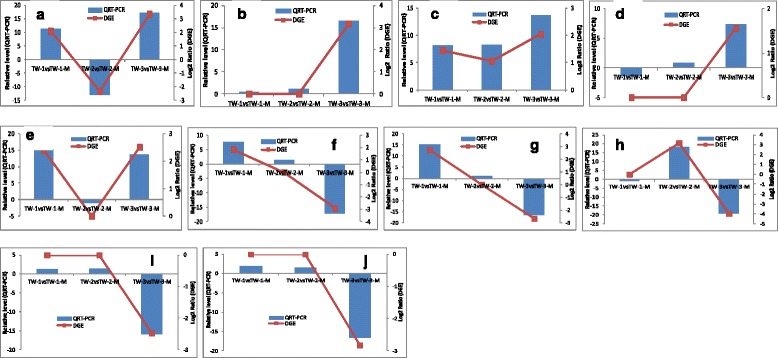
Results of qRT-PCR on 10 genes. **a** Glyma05g31370; **b** Glyma01g12970; **c** Glyma06g02040; **d** Glyma15g03650; **e** Glyma03g41920; **f** Glyma08g08620; **g** Glyma08g14630; **h** Glyma13g22060; **i** Glyma13g30210; **j** Glyma17g34800. Expression data from qRT-PCR basically corroborated the data from Solexa RNA-seq analysis, with both methods yielding the same expression trends

To certify the reliability of the Solexa/Illumina sequencing technology, 10 genes were selected for qRT-PCR assays. The soybean *ACT11* gene was used as an internal control. Although the qRT-PCR expression data was not very consistent with the data from the Solexa RNA-seq analysis, both methods yielded the same expression trends (Fig. 7).

## Discussion

In this study, numerous genes showed different expression levels between TW-1-M and TW-1 mutants. These expression differences were analyzed by RNA-Seq, a fully quantitative method for gene expression evolution [30], providing a new platform to understand the relationships between germination processes and regulatory mechanisms. In our experiments, the number of up-regulated genes was significantly higher than the number of down-regulated genes in TW-1-M, which indicated that most of the genes related to the better germination process and regulatory mechanism were up-regulated. Based on the detailed analysis of high germination-related functionally annotated genes and pathways, genes with the greatest significant differences in expression or abundance were found. These genes were mainly involved in anti-stress, plant hormones, reactive oxygen species and energy metabolism processes. These results were partly consistent with the results from other plants, such as wheat [19], garden pea [31], Arabidopsis [32] and rice [33]. According to these reports [19, 31–33], the genes related to plant hormones and reactive oxygen species might play key roles in seed germination. No report was found on the transcriptomes of LPA crops, especially during the germination process. However, in LPA maize, the free radicals content increased and the seed antioxidation ability decreased because of the reduction in phytate [34], indicating that the genes with antioxidation abilities might be responsible for seed germination rates in LPA crops.

### DGE responses to stress

Based on the changes in the external environment, soybean seeds could use different mechanisms to cope with many biotic and abiotic stresses during germination [35]. In this research, we found many GO functional categories related to abiotic stresses, such as response to salt stress, response to stress and response to heat, even though we performed all of the experiments under the same environmental conditions. These two LPA mutants had a different regulatory response mechanism to the germination environment although their seeds having the same phytate levels and mutated gene. Ten other transcription factors with high levels of differential expression were identified by the DGE analysis in TW-1. These could also participate in the regulation of genes involved in responding to stress and the seed germination process. The high expression level of these genes in the mutant TW-1 might suggest that TW-1 seeds have a different ability to overcome stresses comparing with mutant TW-1-M during the germination stage.

### DGE responses to plant hormones

Seed germination is controlled by both intrinsic and environmental cues, which are mainly regulated by two antagonistic phytohormones, abscisic acid (ABA) and gibberellin (GA) [20]. GA promotes seed germination, whereas ABA has a contrary effect [36]. In our research, we also found three candidate genes, one gibberellin 2-beta-dioxygenase, one gibberellin-regulated protein and ABA receptor *PYL12*, involved in GA and ABA metabolism and signal transduction (Table 3). Gibberellin-regulated protein may function at hormonal-controlled steps of development such as seed germination, flowering and seed maturation [37]. *GA2ox* is responsible for seed dormancy and germination during dark imbibition [38]. These two genes were up-regulated in TW-1-M. *PYLs* function as ABA receptors in the ABA signaling pathway [39], and *PYLs-*mediated ABA signaling could play a crucial role in favoring stress adaptation and growth development for plants [40]. In our results, Gibberellin-regulated protein had the greatest expression abundance, especially in TW-1-M, which could be responsible for TW-1-M’s high germination.

Another plant hormone, ethylene, which participates in the regulation of GA and ABA, could also be responsible for seed germination [41]. In our study, we also found some ethylene regulatory and biosynthesis genes (six ethylene-responsive transcription factors and three 1-aminocyclopropane-1-carboxylate oxidases), which had high expression abundances in the six constructed libraries. All ethylene-responsive transcription factors were down-regulated in TW-1-M, three 1-aminocyclopropane-1-carboxylate oxidases genes were up-regulated in TW-1-M.

### DGE responses to reactive oxygen species

The successful execution of a germination program depends greatly on the seed oxidative homeostasis [26]. Many functional genes and pathways involved in the soybean oxidative process were identified. These genes maintain oxidative balances and reduce oxidative damage to a wide range of cellular components, including DNA, proteins and lipids, and maintain the seeds’ ability to germinate [42–44]. The ascorbate peroxidase gene and L-ascorbate oxidase gene, with their high expression abundances in TW-1-M, play an important role in the regulation of the oxidative state, protecting seeds and maintaining their vigor in mature drying seeds as well as during the early stages of germination [25, 45]. The most differentially expressed gene, Cytochrome P450, were found in both TW-1 and TW-1-M. The P450 family is a large and diverse group of isozymes that mediate a diverse array of oxidative reactions [46]. Polyphenol oxidase A1 was highly expressed in TW-1-M, revealing a vital defense function and protective role in the sensitive early phase of germination and seeding development [47]. The enzyme catalase, which has been employed to determine seed viability [48], was also identified as highly expressed in mutant TW-1. Some DEGs related to flavone metabolism were identified, such as 2-hydroxyisoflavanone synthase, an isoflavone reductase homolog, isoflavone 2’-hydroxylase and isoflavone reductase. These genes are involved in isoflavone biosynthesis, and isoflavone is regarded as an anti-oxidative compound in soybean seeds.

### DGE responses to energy metabolism

Respiration and energy production play key roles in whole seed germination [19]. In this research, some highly expressed and enriched genes related to carbohydrate biosynthesis and metabolism pathways were found, such as glycosyltransferase, glucanase, galactinol synthase, and glucose. These genes might provide energy, translate signaling and be involved in the anti-oxidative process during seed germination.

We also found some embryogenesis abundant protein genes were high expressed in mutant TW-1. Although we compared the transcripts of soybean LPA mutants, we did not find any DEGs related to the phytate metabolic process. This result indicated that these two mutants used the same phytate metabolic pathway in seeds during the germination stage. We identified some candidate genes that might strongly influence seed germination in TW-1-M. However, we still need to perform a genetic analysis and gene mapping to clone these new genes. Further research will help understand the differences in seed germination between the two LPA mutants.

## Conclusions

Improving the seed germination trait of LPA crops is an important goal in crop breeding programs. The gene expression profiling of LPA soybean mutants should provide a substantial contribution to understand the germination mechanism in LPA crops.

In this study, 3,950-9,245 DEGs were identified in each contrast libraries, with TW-1-M having the similar up- and down- regulated DEGs with TW-1. TW-1-M and TW-1 displayed many differentially expressed transcripts involved in seed germination, and DEGs from the seed germination process were mainly related to the ethylene-mediated signaling pathway, oxidation-reduction, the abscisic acid-mediated signaling pathway, response to hormone, ethylene biosynthetic process, ethylene metabolic process, regulation of hormone levels, and oxidation-reduction process, regulation of flavonoid biosynthetic process and regulation of abscisic acid-activated signaling pathway. In total, 2457 DEGs involved in the functional categories above were identified. Twenty-two genes with 20 biological functions were most differentially expressed in high germination-related metabolic or signaling pathways. Fifty-seven genes with 36 biological functions had the greatest expression abundance in germination-related pathways. TW-1-M showed high gene expression in anti-oxidation, GA biosynthesis, stress response and energy metabolism processes, but low gene expression levels in ethylene synthesis during seed germination. The differences in these biological processes between the two LPA mutants could provide a molecular basis for the difference in the seed germination rate.

The findings of this research will allow us to further understand the molecular mechanisms of seed germination in LPA crops. These can also be used as an important resource for the genetic analyses of LPA crop germination traits. Our work suggested that expression diversification of plant hormone- and reactive oxygen species-related genes might strongly contribute to the successful germination in TW-1-M.

## Additional files

Additional file 1: Primers for the qRT-PCR of the 10 tested genes. (DOCX 14 kb)
Additional file 2: A table with the FPKM value of each soybean gene in each of the 18 libraries. (XLSX 5827 kb)
Additional file 3: All DEGs when comparing each germination stage individually in two LPA mutants. (XLSX 6334 kb)
Additional file 4: Genes expressed specifically in three contrast libraries. (XLSX 106 kb)
Additional file 5: Enriched GO terms in the TW-1-M and TW-1 libraries. (XLSX 1127 kb)
Additional file 6: Enriched KEGG pathways in the TW-1-M and TW-1 libraries. (XLSX 293 kb)
Additional file 7: DEGs in each biological process category related to seed germination. (XLSX 83 kb)
Additional file 8: Most differentially expressed genes identified by analysis of DEGs in TW-1-M relative to TW-1. (XLSX 74 kb)
Additional file 9: Special DEGs with FPKM value >100 in each contrast libraries. (XLSX 191 kb)

## Abbreviations

ABA: Abscisic acid
DEGs: Differentially expressed genes
DGE: Digital gene expression
FPKM: Fragment/Kb/million
GA: Gibberellin
LPA: Low phytic acid

## References

[1] Wang ZF, Wang JF, Bao YM, Wang FH, Zhang HS (2010). Quantitative trait loci analysis for rice seed vigor during the germination stage. J Zhejiang Univ Sci B.

[2] Hayashi E, Aoyama N, Still DW (2008). Quantitative trait loci associated with lettuce seed germination under different temperature and light environments. Genome.

[3] Raboy V, Young KA, Dorsch JA, Cook A (2001). Genetics and breeding of seed phosphorus and phytic acid. J Plant Physiol.

[4] Mullaney EJ, Daly CB, Ullah AHJ (2000). Advances in phytase research. Adv Appl Microbiol.

[5] Sharley A, Chapra S, Wedepohl R, Sims J, Daniel T, Reddy K (2008). Managing agricultural phosphorus for protection of surface waters-issues and options. J Environ Qual.

[6] Raboy V (2001). Seeds for a better future: ‘low phytate’ grains help to overcome malnutrition and reduce pollution. Trends Plant Sci.

[7] Nunes ACS, Vianna GR, Cuneo F, Amaya-Farfán J, de Capdeville G, Rech EL, Aragão FJL (2006). RNAi-mediated silencing of the myo-inositol-1-phosphate synthase gene (GmMIPS1) in transgenic soybean inhibited seed development and reduced phytate content. Planta.

[8] Yuan FJ, Zhao HJ, Ren XL, Zhu SL, Fu XJ, Shu QY (2007). Generation and characterization of two novel low phytate mutations in soybean (Glycine max L. Merr.). Theor Appl Genet.

[9] Shi J, Wang H, Wu Y, Hazebroek J, Meeley RB, Ertl DS (2003). The maize low-phytic acid mutant lpa2 is caused by mutation in an inositol phosphate kinase gene. Plant Physiol.

[10] Ali N, Paul S, Gayen D, Sarkar SN, Datta SK, Datta K (2013). RNAi mediated down regulation of myo-inositol-3-phosphate synthase to generate low phytate rice. Rice.

[11] Li WX, Zhao HJ, Pang WQ, Cui HR, Poirier Y, Shu QY (2014). Seed specific silencing of OsMRP5 reduces seed phytic acid and weight in rice. Transgenic Res.

[12] Raboy V (2009). Approaches and challenges to engineering seed phytate and total phosphorus. Plant Sci.

[13] Meis SJ, Fehr WR, Schnebly SR (2003). Seed source effect on field emergence of soybean lines with reduced phytate and raffnose saccharides. Crop Sci.

[14] Oltmans SE, Fehr WR, Welke GA, Raboy V (2005). Agronomic and seed traits of soybean lines with low-phytate phosphorus. Crop Sci.

[15] Spear JD, Fehr WR (2007). Genetic Improvement of Seedling Emergence of Soybean Lines with Low Phytate. Crop Sci.

[16] Bregitzer P, Raboy V (2006). Effects of four independent low-phytate mutations on barley agronomic performance. Crop Sci.

[17] Rutger JN, Raboy V, Moldenhauer KAK, Bryant RJ, Lee FN, Gibbons JW (2004). Registration of KBNT lpa1-1 low phytic acid germplasm of rice. Crop Sci.

[18] Zhao HJ, Liu QL, Ren XL, Wu DX, Shu QY (2008). Gene identification and allele-specific marker development for two allelic low phytic acid mutations in rice (Oryza sativa L.). Mol Breed.

[19] He M, Zhu C, Dong K, Zhang T, Cheng ZW, Li JR, Yan YM. Comparative proteome analysis of embryo and endosperm reveals central differential expression proteins involved in wheat seed germination. BMC Plant Physiol. 2015;15:97–112. 10.1186/s12870-015-0471-z PMC4407426 25888100

[20] Holdsworty MJ, Bentsink L, Soppe WJ (2008). Molecular networks regulating Arabidopsis seed maturation, after-ripening, dormancy and germination. New Phytol.

[21] Goettel W, Xia E, Upchurch R, Wang ML, Chen PY, Charles An YQ (2014). Identification and characterization of transcript polymorphisms in soybean lines varying in oil composition and content. BMC Genomics.

[22] Sun Q, Wang JH, Sun BQ (2007). Advances on seed vigor physiological and genetic mechanisms. Agric Sci China.

[23] Hiroyuki N (2006). Seed germination-the biochemical and molecular mechanisma. Breed Sci.

[24] Wang LK, Feng ZX, Wang X, Wang XW, Zhang XG (2010). DEGseq: an R package for identifying differentially expressed genes from RNA-seq data. Bioinformatics.

[25] Yuan FJ, Zhu DH, Tan YY, Dong DK, Fu XJ, Zhu SL, Li BQ, Shu QY (2012). Identification and characterization of the soybean IPK1 ortholog of a low phytic acid mutant reveals an exon-excluding splice-site mutation. Theor Appl Genet.

[26] Chen CM, Twito S, Miller G. New cross talk between ROS, ABA and auxin controlling seed maturation and germination unraveled in APX6 deficient Arabidopsis seeds. Plant signaling behavior. 2014, 9(12):e976489. http://dx.doi.org/10.4161/15592324.2014.976489. 10.4161/15592324.2014.976489 PMC4622622 25482750

[27] Jones SI, Gonzalez DO, Vodkin LO (2010). Flux of transcript patterns during soybean seed development. BMC Genomics.

[28] Nestor CB, Matilla AJ, Cristina GR, Maria DCRG (2015). ABA-stimulated SoDOG1 expression is after-ripening inhibited during early imbibition of germinating Sisymbrium offcinale seeds. Physiol Plant.

[29] Knaehisa M, Goto S, Kawashima S, Okuno Y, Hattori M (2004). The KEGG resource for deciphering the genome. Nucleic Acids Res.

[30] Wu X, Walker MG, Lou J, Wei L (2005). GAB server: EST-based digital gene expression profiling. Nucleic Acids Res.

[31] Kranner I, Roach T, Beckett RP, Whitaker C, Minibayeva FV (2010). Extracellular production of reactive oxygen species during seed germination and early seedling growth in *Pisum sativum*. J Plant Physiol.

[32] Xi W, Liu C, Hou X, Yu H (2010). MOTHER OF TR AND TFL1 regulates seed germination through a negative feedback loop modulation ABA signaling in Arabidopsis. Plant Cell.

[33] He D, Han C, Yang P (2011). Gene expression profile changes in germinating rice. J Integr Plant Biol.

[34] Doria E, Galleschi L, Calucci L, Pinzino C, Pilu R, Cassami E, Nielsen E (2009). Phytic acid prevents oxidative stress in seeds: evidence from a maize (Zea mays L.) low phytic acid mutant. J Exp Bot.

[35] Finnie C, Melchior S, Roepstorff P, Svensson B (2002). Proteome analsyis of grain filling and seed maturation in barley. Plant Physiol.

[36] Helenius AES (1997). Trombetta: Calnexin, calreticulin and the folding of glycoproteins. Trends Cell Biol.

[37] Yamauchi Y, Ogawa M, Kuwahara A, Hanada A, Kamiya Y (2004). Activation of gibberellin biosynthesis ans response pathways by low temperature during imbibitionof Arabidopsis thaliana seeds. Plant Cell.

[38] Yamauchi Y, Takeda-Kamiya N, Hanada A, Ogawa M, Kuwahara A (2007). Contribution of gibberellin deactivation by AtGA2ox2 to suppression of germination of dark-imbibed Arabidopsis thaliana seeds. Plant Cell Physiol.

[39] Fujii H, Chinnusamy V, Rodrigues A, Rubio S, Antoni R, Park SY (2009). In vitro reconstitution of an abscisic acid signalling pathway. Nature.

[40] Zhang XL, Jiang L, Xin Q, Liu Y, Tan JX, Chen ZZ (2015). Structural basis and functions of abscisic acid receptors PYLs. Plant Sci.

[41] Kucera B, Cohn MA, Leubner-Metzger G (2005). Plant hormone interactions during seed dormancy release and germination. Seed Sci Res.

[42] Parkhey S, Naithani SC, Keshavkant S (2012). ROS production and lipid catabolism in desiccating shorea robusta seeds during aging. Plant Physiol Biochem.

[43] Bailly C, EI-Maarouf-Bouteau H, Corbineau F (2008). From intracellular signaling networks to cell death: the dual role of reactive oxygen species in seed physiology. C R Biol.

[44] Chen Q, Yang L, Ahmad P, Wan X, Hu X (2011). Preteomic profiling and redox status alteration of recalcitrant tea seed in response to desiccation. Planta.

[45] Chen C, Letnik I, Hacham Y, Dobrev P, Ben-Daniel BH, Vankova R, Amir R, Miller G (2014). Ascorbate peroxidase 6 protects Arabidopsis thaliana desiccating and germinating seeds from stress and mediates crosstalk between ROS, ABA and auxin. Plant Physiol.

[46] Chen H, Wang FW, Dong YY, Wang N, Sun YP, Li XY, Liu L, Fan XD, Yin HL, Jing YY, Zhang XY, Li YL, Chen G, Li HY (2012). Sequence mining and transcript profiling to expore differentially wxpressed genes associated with lipid biosynthesis during soybean seed development. Plant Biol.

[47] Sahbaz R, Lieberei R, Aniszewski T (2009). Polyphenol oxidase (PPO, catecholase) activity during germination and early seedling growth of cicer milkvetch (Astragalus cicer L.). J Appl Bot Food Qual.

[48] Ayse AK, Yucel E, Sezgin A (2012). Relationship between seed germination and catalase enzyme activity of Abies taxa from Turkey. J For Fac Kastamonu Univ.

