# Supplementary material for: Whole genome-wide transcript profiling to identify differentially expressed genes associated with seed field emergence in two soybean low phytate mutants
Source: BMC Plant Biol. 2017 Jan 18;17:16. doi: 10.1186/s12870-016-0953-7 (PMC5242038; doi:10.1186/s12870-016-0953-7)
Supplement: Additional file 1: — Primers for the qRT-PCR of the 10 tested genes. (DOCX 14 kb) [file 12870_2016_953_MOESM1_ESM.docx]

Table 4 Primers of qRT-PCR

| Gene | Ta Opt | Size | Sequence(5’-3’) | Amplification effciencies of primer |
| --- | --- | --- | --- | --- |
| Glyma05g31370 | 49.8 | 146 | F: TCGACTAGAAACGGTATT | 93% |
|  |  |  | R: GAAAGCGACTCTTGAACT |  |
| Glyma01g12970 | 52.3 | 251 | F: TAGCTCAGCGAATCCACG | 98% |
|  |  |  | R: CAGCCTATGTTCACCACC |  |
| Glyma06g02040 | 47.1 | 158 | F: GTCGCTTGGTCCTGAATA | 96% |
|  |  |  | R: AAGTCTGTGCCTTTGTGA |  |
| Glyma15g03650 | 50.1 | 148 | F: AGCCTGTGACTGGTGCTT | 95% |
|  |  |  | R: GTCGGTCCAATCGGGTTA |  |
| Glyma03g41920 | 48.9 | 125 | F: CCCAACTATTCACTGTCC | 95% |
|  |  |  | R: CCTTGCATTTGCACCTTA |  |
| Glyma08g08620 | 57.5 | 275 | F: CCACCACGGAAGACGACA | 99% |
|  |  |  | R: GCACCAATCCAGCCCTTT |  |
| Glyma08g14630 | 48.9 | 140 | F: CACTGGACAAGCGAGATG | 96% |
|  |  |  | R: TTGGTCAAAGTCCGTTCC |  |
| Glyma13g22060 | 49 | 152 | F: GGTGAGAATCATCCGTTTA | 95% |
|  |  |  | R: ATTTGCCTACTAAGAGTGG |  |
| Glyma13g30210 | 55.9 | 151 | F: AGAAGCAGCCAACGCCACC | 95% |
|  |  |  | R: GACGGAGAAGCACTGAGAAGGA |  |
| Glyma17g34800 | 48.8 | 264 | F: CCAAGAACCCAATTACAAAA | 94% |
|  |  |  | R: GAGTTTACGGCGGATTGA |  |
| *Act11* | 50.2 | 213 | F: CAACCCAAAGGTCAACAG | 96% |
|  |  |  | R: CAGCGAGATCCAAACGAA |  |
